# Baculovirus infection induces disruption of the nuclear lamina

**DOI:** 10.1038/s41598-017-08437-5

**Published:** 2017-08-10

**Authors:** Xiaomei Zhang, Kaiyan Xu, Denghui Wei, Wenbi Wu, Kai Yang, Meijin Yuan

**Affiliations:** State Key Laboratory of Biocontrol, Sun Yat-sen University, Guangzhou, 510275 China

## Abstract

Baculovirus nucleocapsids egress from the nucleus primarily via budding at the nuclear membrane. The nuclear lamina underlying the nuclear membrane represents a substantial barrier to nuclear egress. Whether the nuclear lamina undergoes disruption during baculovirus infection remains unknown. In this report, we generated a clonal cell line, Sf9-L, that stably expresses GFP-tagged *Drosophila* lamin B. GFP autofluorescence colocalized with immunofluorescent anti-lamin B at the nuclear rim of Sf9-L cells, indicating GFP-lamin B was incorporated into the nuclear lamina. Meanwhile, virus was able to replicate normally in Sf9-L cells. Next, we investigated alterations to the nuclear lamina during baculovirus infection in Sf9-L cells. A portion of GFP-lamin B localized diffusely at the nuclear rim, and some GFP-lamin B was redistributed within the nucleus during the late phase of infection, suggesting the nuclear lamina was partially disrupted. Immunoelectron microscopy revealed associations between GFP-lamin B and the edges of the electron-dense stromal mattes of the virogenic stroma, intranuclear microvesicles, and ODV envelopes and nucleocapsids within the nucleus, indicating the release of some GFP-lamin B from the nuclear lamina. Additionally, GFP-lamin B phosphorylation increased upon infection. Based on these data, baculovirus infection induced lamin B phosphorylation and disruption of the nuclear lamina.

## Introduction

The nuclear envelope (NE) consists of the inner nuclear membrane (INM) and outer nuclear membrane (ONM), which are separated by the perinuclear space and spanned by nuclear pore complexes (NPCs)^1^. Metazoan NEs possess an additional feature, the nuclear lamina, a rigid protein meshwork underlying the nucleoplasmic face of the INM^2, 3^. The major components of the nuclear lamina are type V intermediate filament proteins known as lamins, which are grouped into two categories: A-type lamins, including lamin A and lamin C, and B-type lamins, including lamin B1, lamin B2 and lamin B3. While most vertebrates express one A-type lamin and two B-type lamins, invertebrates possess only a single B-type lamin gene with certain exceptions, such as *Drosophila*, which expresses one B-type lamin (lamin Dm_0_) and one A-type lamin (lamin C)^2^. INM-associated B-type lamin is the fundamental building block of the lamina and is essential for nuclear shape maintenance, whereas nucleoplasm-associated A-type lamin provides more specialized functions and contributes to nuclear stiffness^4^. Like all intermediate filaments, lamins comprise a central α-helical rod domain flanked by globular head and tail domains. Lamins dimerize via coiled-coil interactions between rod domains and associate in a head-to-tail fashion to form longer filaments^5^. Lamin filaments pack tightly to form regular cross-connections with an average crossover spacing of 5 nm^6^. The lamin-assembled lamina plays important roles in maintaining the architecture of the NE and regulating normal cell physiology^1^. This rigid meshwork also represents a natural barrier against most DNA viruses^4^.

Despite its relative insolubility and structural rigidity, the lamina is a dynamic structure. During mitosis and apoptosis, the lamina is disassembled via site-specific lamin phosphorylation mediated by different kinases^7, 8^. During herpesvirus infection, the lamina is disrupted locally through multiple mechanisms to ensure the efficient production of viral progeny^9, 10^. While herpesvirus genome packaging and capsid formation occur in the nucleus, continuing maturation via the addition of tegument proteins as well as final envelopment occurs in the cytoplasm. Thus, herpesvirus nucleocapsids must cross the NE to access the final maturation compartment^10, 11^. Because these nucleocapsids are too large to traverse nuclear pores or the crossover spacing of the nuclear lamina, herpesviruses dissolve the nuclear lamina through a kinase-mediated phosphorylation mechanism and bud at the INM for nuclear egress^4, 9^. Both viral and cellular kinases are involved in the phosphorylation of lamins, which then mediate partial dismantling of the nuclear lamina. Two conserved herpesvirus proteins, designated pUL31 and pUL34 for *Alphaherpesvirinae*, are required for this process^9, 12^.

The family *Baculoviridae* is a diverse group of insect-specific viruses with circular double-stranded DNA genomes packaged into rod-shaped, enveloped nucleocapsids^13, 14^. *Autographa californica multiple nucleopolyhedrovirus* (AcMNPV) is the archetype species of the genus *Alphabaculovirus*. Members of AcMNPV undergo a biphasic replication cycle that typically involves the production of two morphologically distinct virion phenotypes: the budded virion (BV) and the occlusion-derived virion (ODV)^15, 16^. The major differences between BVs and ODVs lie in the origin and composition of their envelopes, which parallel their different roles in the baculovirus life cycle^17–20^. Like herpesvirus, the assembly of baculovirus progeny nucleocapsids occurs in the nuclei of infected cells. During the early phase of infection, nucleocapsids egress through the nuclear membrane, migrate across the cytosol, and acquire their envelopes from plasma membrane decorated with viral proteins to form BVs which are responsible for spreading infections within susceptible insect tissues or among cells in culture^21, 22^. Later in infection, the nucleocapsids are retained within the nucleus and acquire their envelopes from virus-induced intranuclear microvesicles to form ODVs which can initiate primary infection in the midgut epithelium of infected insects and thus are required for the horizontal transmission of infection among insect hosts^15, 23^.

Although several routes of baculovirus nucleocapsid egress from the nucleus have been proposed, including via nuclear pores, migration into or through the endoplasmic reticulum, or passage through discontinuities in the NE, the most common method of egress observed in electron microscopy studies of NPV involves a budding process at the nuclear membrane^24^. Nucleocapsids have been observed to bud through the INM into an enlarged perinuclear space^25^. Retention of ‘enveloped’ nucleocapsids in the perinuclear space was observed in cells which were transfected with a mutant *Bombyx mori* NPV with its open reading frame (ORF) 67 (*bm67*) deleted^26^. Baculovirus nucleocapsids are 40–70 nm in diameter and 250–400 nm in length^23^ and thus are too large to pass through the crossover spacing of the nuclear lamina. Therefore, baculovirus nucleocapsids should modify the nuclear lamina to gain access to the budding site at the nuclear membrane.

Although the nuclear egress of baculoviruses is similar to that of herpesviruses, whether the nuclear lamina is disrupted during baculovirus infection remains unknown. In this study, we generated a *Spodoptera frugiperda*-derived IPLB-Sf21-AE clonal isolate 9 (Sf9)-L clonal cell line stably expressing enhanced green fluorescent protein (GFP)-tagged *Drosophila* lamin B (GFP-lamin B) and studied alterations to the nuclear lamina in the context of baculovirus infection. Some GFP-lamin B was redistributed in the ring zone within the nuclei of Sf9-L cells and associated with virions during baculovirus infection, indicating partial disruption of the nuclear lamina; in contrast, mock-infected cells exhibited specific nuclear rim distribution. Furthermore, GFP-lamin B phosphorylation increased upon infection. Thus, we provide the first evidence of baculovirus infection-induced lamin B phosphorylation and disruption of the nuclear lamina.

## Results

### Generation of a clonal cell line stably expressing GFP-tagged lamin B

**Figure 1:**
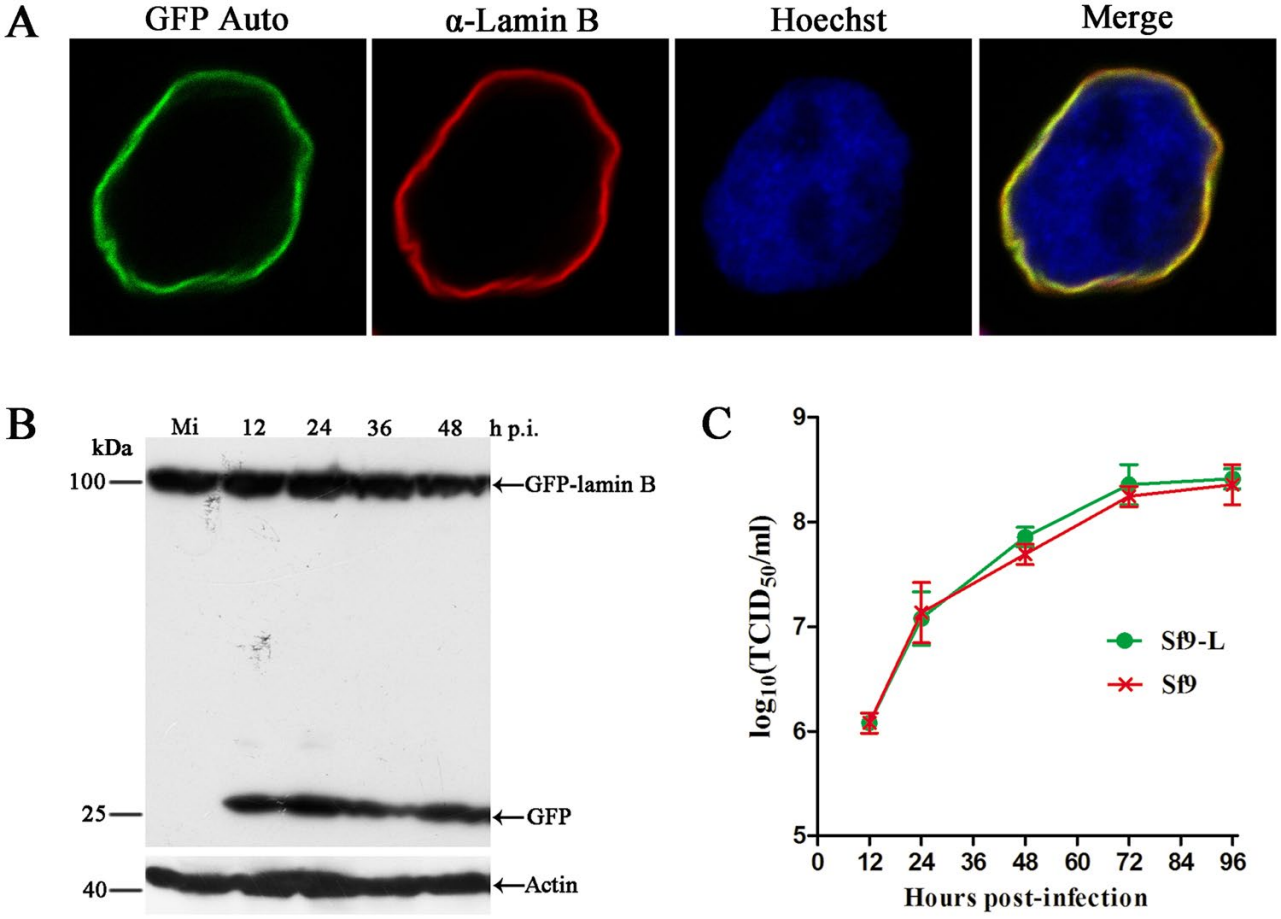
Generation of the Sf9-L clonal cell line. (**A**) Subcellular localization of lamin B in Sf9-L cells. The cells were fixed, permeabilized, stained with the mouse monoclonal antibody ADL67, and visualized with donkey anti-mouse IgG conjugated to Alexa Fluor 555 (red) as the secondary antibody to detect both native lamin B and chimeric GFP-lamin B. Chimeric GFP-lamin B was detected by visualizing GFP autofluorescence (GFP-Auto, green). Hoechst 33342 was used to identify the nuclei and DNA-rich regions (blue). (**B**) Time course analysis of total GFP-lamin B. Sf9-L cells were mock-infected or infected with vAcWT at an MOI of 10. At the indicated time points, the cells were collected, resolved by SDS-10% PAGE, and subjected to Western blotting with a mouse monoclonal antibody against GFP and an anti-actin antibody as a loading control. Mi, mock-infected Sf9-L cells. (**C**) Viral replication analysis in Sf9-L and Sf9 cells. Sf9-L and Sf9 cells were infected with vAcWT-mCh at an MOI of 5, and the supernatants were harvested at the selected time points. The titers were determined using TCID_50_ assays. Each data point represents the average of three independent infections. The error bars represent the standard deviations.

The nuclear lamina represents a natural barrier against most DNA viruses when progeny viral nucleocapsids egress from the nucleus to the cytoplasm of infected cells. Herpesviruses breach this barrier by recruiting cellular and viral kinases to phosphorylate lamins, which leads to disruption of the nuclear lamina^9^. To investigate whether any alterations to the nuclear lamina occur during baculovirus infection, we sought to generate an Sf9 cell line stably expressing GFP-tagged lamin B to analyze the nuclear lamina with respect to its major component, lamin B, in AcMNPV-infected Sf9 cells. The full-length sequence of Sf9 lamin B was not available due to the lack of a reference genome for Sf9 cells at the beginning of our study. *Drosophila* lamin B has been well characterized and associates with the nuclear lamina when expressed in Sf9 cells^27^. In addition, an antibody against *Drosophila* lamin B (ADL67 antiserum) recognizes the Sf9 nuclear lamina^28, 29^. Thus, *Drosophila lamin B* was fused to a GFP tag-coding sequence at its 3′ terminus (GFP-lamin B) to monitor alterations to the nuclear lamina. The coding sequence for GFP-lamin B was cloned into pIB/V5-His. Sf9 cells were transfected with the resulting construct, pIB-GFP:LmnB, and grown in culture medium containing 60 μg/ml blasticidin to select for chimeric protein expression. To obtain more homogeneous GFP-lamin B expression for subsequent experiments, a clonal cell line stably expressing GFP-lamin B (Sf9-L) was isolated using Millicell inserts. Confocal microscopy revealed a nuclear rim fluorescence pattern in Sf9-L cells, and GFP autofluorescence colocalized with the immunofluorescence signal generated by the antibody ADL67, which recognized both native lamin B and chimeric GFP-lamin B (Fig. 1A). Based on these results, GFP-lamin B was correctly incorporated into the nuclear lamina.

To further confirm chimeric protein expression in Sf9-L cells, equivalent amounts of mock-infected and vAcWT-infected Sf9-L cells were harvested at the indicated time points and analyzed by Western blotting with a mouse monoclonal anti-GFP antibody. Cellular actin staining was performed to confirm equivalent protein loading. As shown in Fig. 1B, an immunoreactive band of approximately 100 kDa corresponding to the predicted molecular mass of full-size GFP-lamin B was detected in both mock-infected and virus-infected cell lysates, indicating GFP-lamin B was expressed as a fusion protein in Sf9-L cells. The GFP-only fragment was detected in virus-infected cells because vAcWT expresses GFP^30^, but not in mock-infected cells (Fig. 1B), indicating the GFP-lamin B fusion protein was not cleaved in Sf9-L cells.

**Figure 2:**
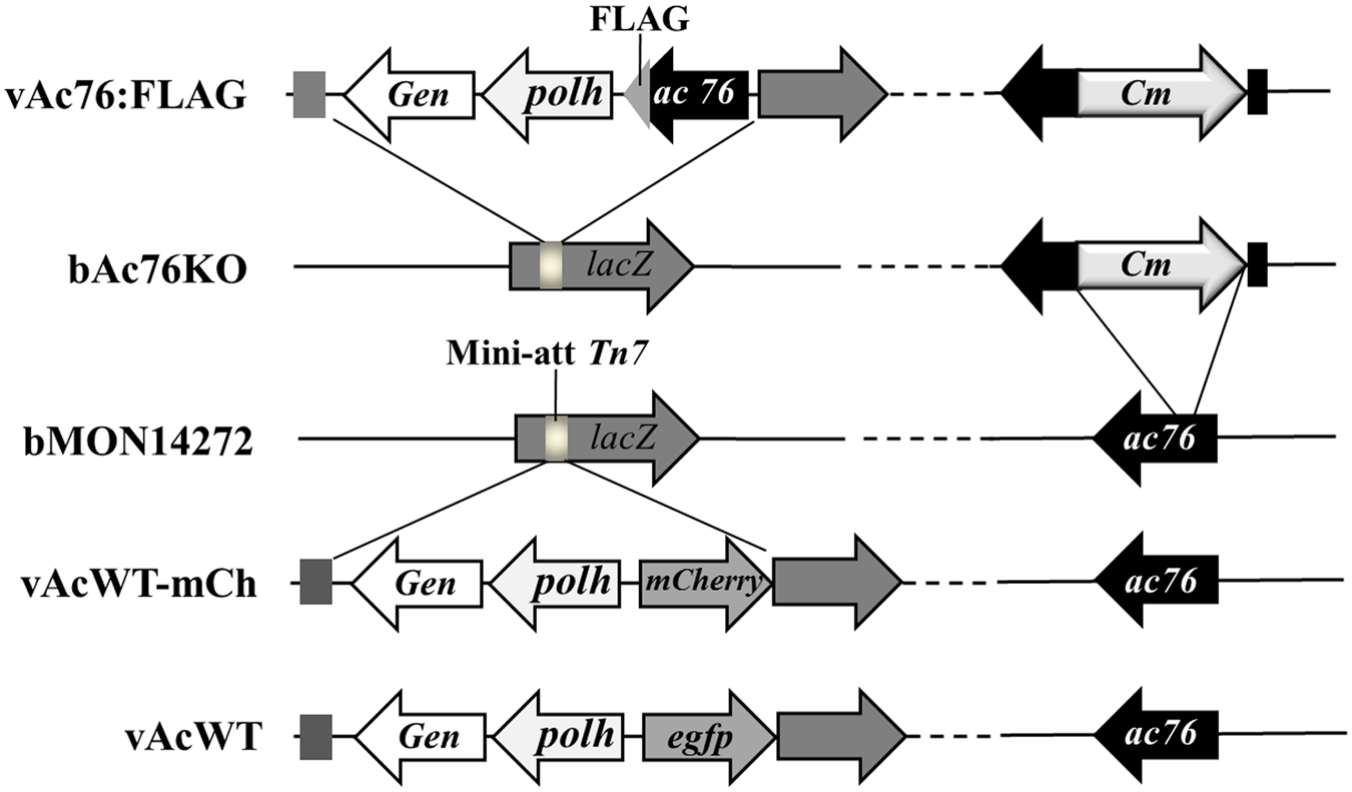
Schematic diagram of the AcMNPV bacmid constructs used in this study. The genes are indicated in a linear representation of the AcMNPV genome. Arrows denote the protein-coding regions and transcription orientations of the genes.

To assess whether the presence of GFP-lamin B affects viral replication, a recombinant wild type virus with an *mCherry fluorescent protein* reporter gene, vAcWT-mCh, was constructed by inserting the *polyhedrin* and *mCherry* genes into the *polyhedrin* locus of the bacmid bMON14272 (Fig. 2). The replication of vAcWT-mCh in Sf9-L and Sf9 cells was compared in a 50% tissue culture infective dose (TCID_50_) endpoint dilution assay. As shown in Fig. 1C, the virus exhibited similar growth kinetics in both cell lines, indicating normal propagation in Sf9-L cells.

Given these results, the Sf9-L cell line is appropriate for studying the fate of lamin B during the course of baculovirus infection.

### The nuclear lamina is partially disrupted during baculovirus infection

To investigate the effects of viral infection on nuclear lamina dynamics, the subcellular localization of GFP-lamin B was analyzed by performing confocal microscopy. Because virus-encoded protein Ac76 associates with the endoplasmic reticulum in the cytoplasm during the early phase of infection, moves into the nucleus and then localizes to the nuclear membrane, intranuclear microvesicles and the ODV envelope during the late phase of infection^29, 31^, this protein was detected as a control to present a rough estimation of localization, particularly in the nucleus. Thus, we constructed an *ac76*-repair virus vAc76:FLAG in which the AcMNPV *ac76* cassette fused to a FLAG tag-coding sequence at the 3′ terminus, as well as the *polyhedrin* gene, was inserted into the *polyhedrin* locus of the *ac76* knockout bacmid bAc76KO^31^ (Fig. 2). The effects of FLAG-tagged Ac76 on viral replication and morphogenesis were assessed in a TCID_50_ endpoint dilution assay and by transmission electron microscope (TEM). vAc76:FLAG titers were similar to those of vAcWT, and cells infected with vAc76:FLAG exhibited characteristics similar to those infected with vAcWT (data not shown), indicating FLAG-tagged Ac76 possessed the same functions as the original Ac76 and rescued the defective vAc76KO phenotype. Thus, we used vAc76:FLAG for subsequent analyses.

**Figure 3:**
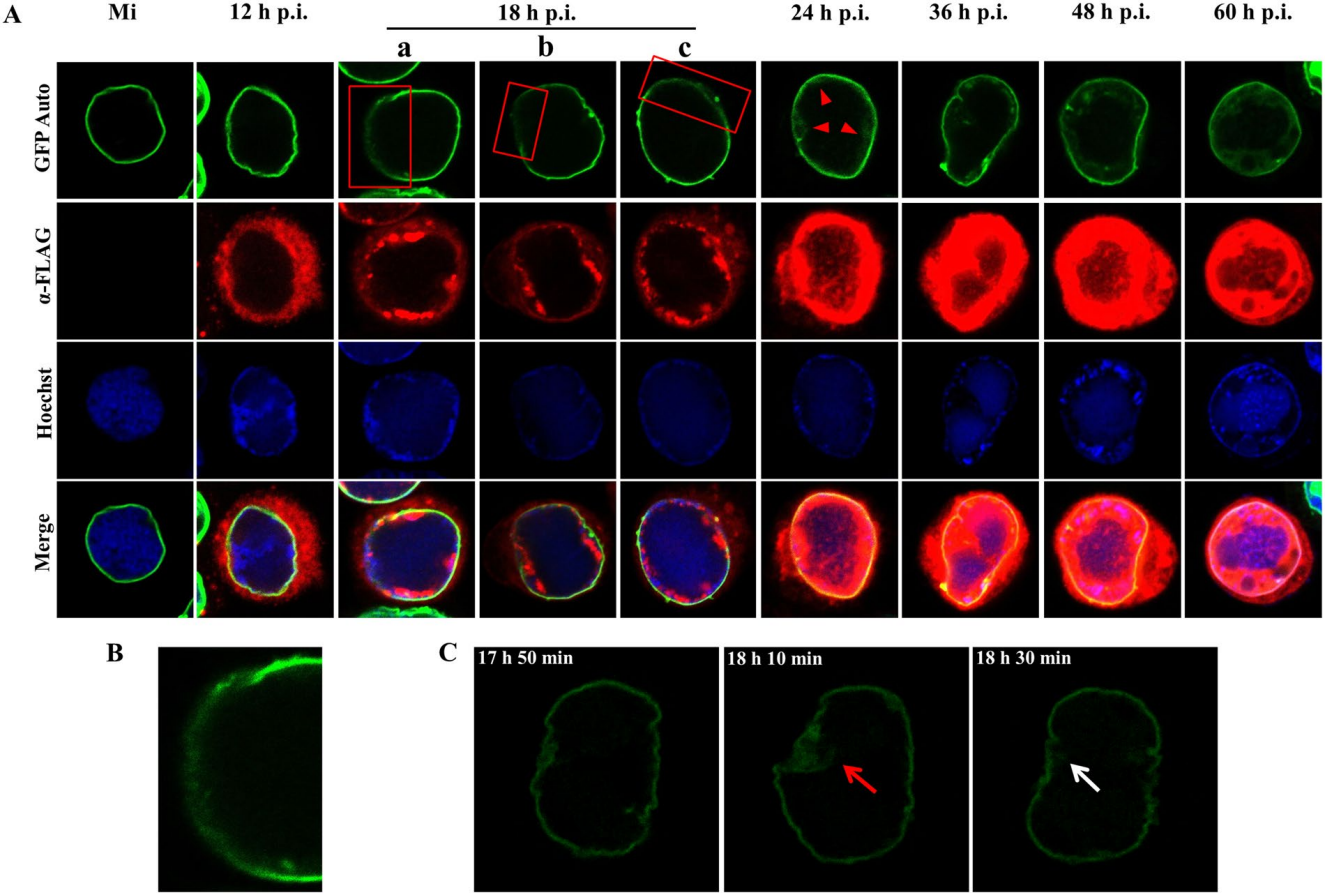
Subcellular localization of GFP-lamin B in Sf9-L cells. (**A**) Immunofluorescence microscopy analysis. Sf9-L cells were mock-infected or infected with vAc76:FLAG at an MOI of 5. At the designated time points p.i., the cells were fixed, permeabilized, stained with a mouse monoclonal anti-FLAG antibody, and visualized with donkey anti-mouse IgG conjugated to Alexa Fluor 555 (red) as the secondary antibody to detect Ac76. GFP-lamin B was detected based on GFP autofluorescence (GFP-Auto, green). Hoechst 33342 was used to identify nuclei and DNA-rich regions (blue). The red rectangles indicate the diffuse localization of a portion of the nuclear rim GFP-lamin B, and the red triangles show the distribution of GFP-lamin B at the periphery of the nucleus. (**B**) Higher magnification image of the boxed region in (**Aa**). (**C**) Live confocal microscopy images of a virus-infected Sf9-L cell between 17 h 50 min and 18 h 30 min p.i. A portion of the nuclear rim GFP-lamin B showed diffuse localization (red arrow) and then recovered (white arrow).

Sf9-L cells were infected with vAc76:FLAG, collected at designated time points and processed for immunofluorescence analyses. GFP autofluorescence was indicative of GFP-lamin B localization, whereas anti-FLAG antibody immunofluorescence revealed the distribution of Ac76 (Fig. 3A). As expected, in mock-infected cells, GFP-lamin B fluorescence uniformly localized to the nuclear rim, and Ac76 fluorescence was not observed. In vAc76:FLAG-infected cells, Ac76 was observed along the outer periphery of the nuclear membrane at 12 h post infection (p.i.), followed by movement into the nuclei and the formation of discrete foci at the nuclear periphery corresponding to the intranuclear ring zone at 18 h p.i., and finally concentration at the ring zone beginning at 24 h p.i., consistent with a previous study^31^. For GFP-lamin B, marked alterations in the distribution pattern were detected. The distribution pattern was similar to that in mock-infected cells at 12 h p.i. However, a portion of the GFP-lamin B at the nuclear rim showed altered localization and seemed diffuse at 18 h p.i. in approximately 67.5% of more than 40 cells observed (Fig. 3A, red rectangles, and Fig. 3B), indicating structural rearrangement of the nuclear lamina. The percentage of cells with the altered localization of nuclear rim GFP-lamin B decreased to 33.3% at 24 h p.i. and 13.2% at 36 h p.i. Live confocal imaging of individual cells captured between 17 h 50 min and 18 h 30 min p.i. revealed the diffuse localization and recovery of a partial area of the nuclear rim GFP-lamin B (Fig. 3C, red arrow and white arrow), suggesting this altered localization of GFP-lamin B at nuclear rim was a dynamic process. By 24 h p.i., GFP-lamin B primarily localized at the nuclear rim but started to accumulate at the nuclear periphery (Fig. 3A, red triangles). GFP-lamin B fluorescence within the nucleus gradually increased as infection progressed. By 60 h p.i., GFP-lamin B within the nucleus became more concentrated and colocalized with Ac76 at the ring zone (Fig. 3A). As the total amount of lamin B did not increase during the course of infection according to the Western blot results and a recent study^32^, these data implied a partial disruption of the nuclear lamina and redistribution of the disassembled GFP-lamin B within the nuclei during baculovirus infection.

### GFP-lamin B is associated with virions in baculovirus-infected Sf9-L cells

**Figure 4:**
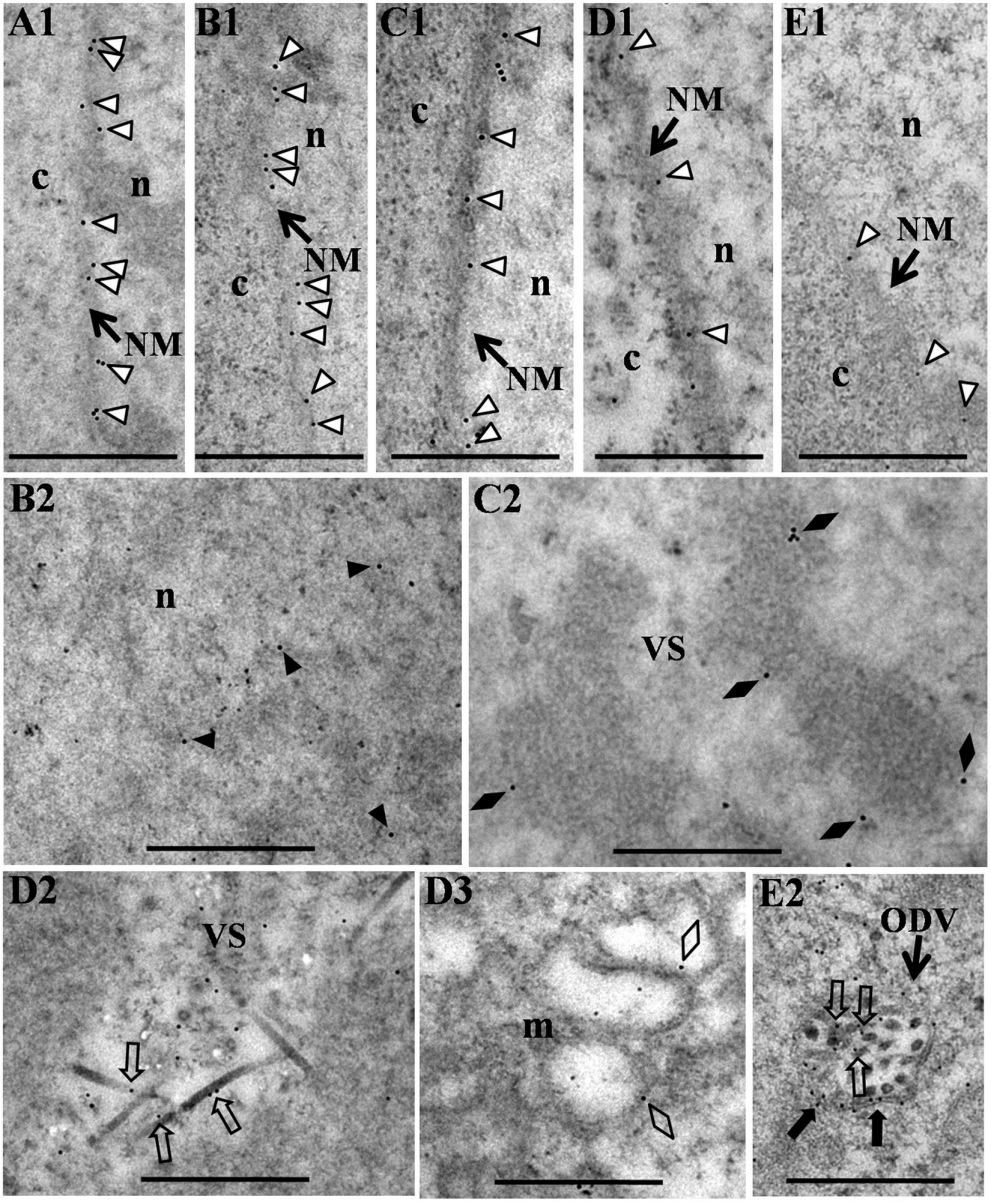
Immunoelectron micrographs showing the distribution of GFP-lamin B in mock- or vAc76:FLAG-infected Sf9-L cells. At the designated time points p.i., the cells were fixed, dehydrated, embedded in LR White resin, and processed for immunogold labeling with a mouse monoclonal anti-GFP antibody. (**A1**) A mock-infected cell. (**B1–E2**) vAc76:FLAG-infected cells at 12 h p.i. (**B1, B2**), 24 h p.i. (**C1, C2**), 36 h p.i. (**D1–D3**) and 48 h p.i. (**E1, E2**). c, cytoplasm; n, nucleus; NM, nuclear membrane; m, microvesicle; VS, virogenic stroma; ODV, occlusion-derived virion; open triangles, labeling of the nuclear lamina; black triangles, labeling of the VS; black diamonds, labeling of the edges of the electron-dense stromal mattes of the VS; open arrows, labeling of the nucleocapsids; open diamonds, labeling of intranuclear microvesicles; black arrows, labeling of the ODV envelopes. Scale bars, 500 nm.

To explore the localization of GFP-lamin B in more detail, Sf9-L cells infected with vAc76:FLAG were collected at the indicated time points and processed with a mouse monoclonal anti-GFP antibody for immunoelectron microscopy (IEM). As expected, gold particles specifically localized to the nuclear lamina in mock-infected cells (Fig. 4A1, open triangles), consistent with the confocal microscopy results (Fig. 3A). At 12 h p.i., the majority of the gold particles labeled the nuclear lamina (Fig. 4B1, open triangles), and a few gold particles were associated with a loose granular material in the nucleoplasm which should be the virogenic stroma (VS) as the VS is manifest as a loose granular matrix at the early phase of infection^24^ (Fig. 4B2, black triangles). Beginning at 24 h p.i., in addition to labeling the nuclear lamina (Fig. 4C1, D1 and E1, open triangles), gold particles accumulated within the nuclei, specifically associated with the edges of the electron-dense stromal mattes of the VS (Fig. 4C2, black diamonds) and the nucleocapsids at the periphery of the stromal mattes (Fig. 4D2, open arrows), or localized to virus-induced intranuclear microvesicles (Fig. 4D3, open diamonds) and the envelopes (Fig. 4E2, black arrows) and nucleocapsids (Fig. 4E2, open arrows) of ODVs in the ring zone. Based on these results, the nuclear lamina was partially disrupted during baculovirus infection, and lamin B that disassociated from the nuclear lamina was redistributed in the nucleus and associated with virions.

### Baculovirus infection induces lamin B phosphorylation in Sf9-L cells

**Figure 5:**
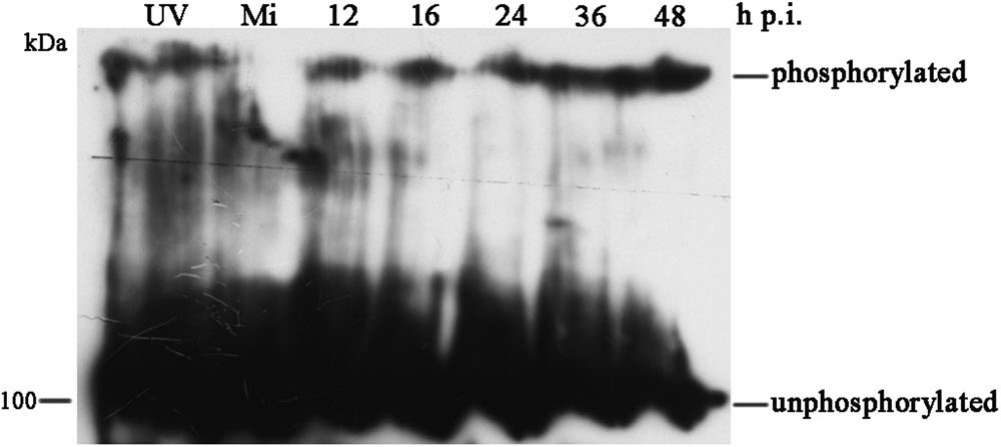
Phosphorylation of GFP-lamin B during baculovirus infection. Sf9-L cells were mock-infected or infected with vAcWT. The cells were harvested at the indicated time points p.i. The proteins were separated by Mn^2+^-Phos-tag SDS-8% PAGE and subjected to Western blotting with a mouse monoclonal anti-GFP antibody. Sf9-L cells exposed to ultraviolet light (UV) for 4 h were used as a positive control. Mi, mock-infected Sf9-L cells.

The disruption of the nuclear lamina that occurs either during herpesvirus infection or during cellular mitosis and apoptosis^7, 9, 33^ correlates with lamin phosphorylation^7, 9, 34^. Since baculovirus infection induces the disruption of the nuclear lamina, we then tested whether lamin B phosphorylation increased in baculovirus-infected cells. Sf9-L cells that were mock-infected or infected with vAcWT were collected at designated time points and resolved by Mn^2+^-Phos-tag SDS-polyacrylamide gel electrophoresis (PAGE), which separates phosphorylated and unphosphorylated proteins based on mobility shift as the polyacrylamide-bound Mn^2+^-Phos-tag preferentially traps the phosphorylated proteins and results in the lower mobility of phosphorylated proteins^35^. These proteins were then subjected to Western blot analysis using an anti-GFP antibody. Cells exposed to ultraviolet light for 4 h to induce apoptosis and lamin B phosphorylation were used as a positive control. As shown in Fig. 5, a major band of approximately 100 kDa corresponding to the predicted molecular mass of full-size unphosphorylated GFP-lamin B was detected in all protein samples. A slower migrating band was detected in the positive control, but not in mock-infected cells, indicating that this band should correspond to phosphorylated GFP-lamin B. The phosphorylated band was detected in virus-infected cell extracts beginning at 12 h p.i. and maintained until 48 h p.i. Thus, baculovirus infection induced lamin B phosphorylation in Sf9-L cells as early as 12 h p.i., suggesting the disruption of the nuclear lamina most likely correlates with lamin B phosphorylation during baculovirus infection.

## Discussion

The lamina meshwork represents a barrier to nucleocapsid budding for some DNA viruses, such as herpesviruses, which breach this barrier by recruiting viral and cellular kinases to phosphorylate lamins and dissolve the lamina^9^. Baculovirus nucleocapsids egress from the nucleus primarily by budding through the nuclear membrane, a process widely documented by electron microscopy^24^. However, whether the nuclear lamina undergoes disruption during the course of baculovirus infection remains unknown. In the present study, we investigated the alterations to the nuclear lamina during AcMNPV infection. Based on several lines of evidence from our work, the nuclear lamina was partially disrupted upon baculovirus infection, the disassembled lamin B was associated with virions and partial disruption of the nuclear lamina most likely correlated with lamin B phosphorylation.

Although antibodies against *Drosophila* lamin B recognize the Sf9 nuclear lamina in immunofluorescence and Western blot analyses^28, 29, 36^, gold particles were barely detectable at the nuclear lamina using IEM with anti-*Drosophila* lamin B in Sf9 cells in our study (data not shown), which is potentially attributable to epitope masking of anti-*Drosophila* lamin B. Thus, we sought to generate a clonal cell line stably expressing GFP-tagged lamin B to investigate lamin B localization based on GFP autofluorescence and the application of commercial GFP antibodies. The full-length sequence of Sf9 lamin B was not yet available due to the lack of a reference genome for Sf9 cells at the beginning of our study, but previous studies have shown an association between *Drosophila* lamin B and the nuclear lamina when expressed in Sf9 cells^27^; meanwhile, the identified phosphorylation sites, including cyclin-dependent kinase 1 (CDK1) recognition sites, protein kinase C (PKC) recognition sites, or even herpesvirus-encoded conserved herpesvirus protein kinases (CHPKs)-mediated phosphorylation sites, are quite conserved among different species of lamins, indicating similar mechanisms of lamin regulation between different lamins and different species^4, 9, 37^. Therefore, GFP-tagged *Drosophila* lamin B was stably expressed in Sf9 cells to monitor lamin B localization and nuclear lamina dynamics during baculovirus infection. GFP-tagged lamins, including A-type and B-type lamins, have previously been used to investigate lamin localization and dynamics, and these chimeric proteins behave similarly to endogenous lamin molecules^38–40^. In this study, the colocalization of GFP autofluorescence with the immunofluorescence signal of the ADL67 antibody, which recognizes both endogenous Sf9 lamin molecules and GFP-lamin B, at the nuclear rim of Sf9-L cells indicated proper incorporation of GFP-lamin B into the nuclear lamina. Additionally, viral titers in Sf9-L and Sf9 cells were comparable, indicating the presence of GFP-lamin B did not disturb viral replication. The above data suggested that the GFP-tagged *Drosophila* lamin B behaved similarly to endogenous Sf9 lamin molecules. Thus, Sf9-L cells stably expressing GFP-lamin B are appropriate for investigating the fate of lamin B during baculovirus infection.

Immunofluorescence and IEM studies of GFP-lamin B provided direct evidences of the partial disruption of the nuclear lamina during baculovirus infection. In contrast to the specific nuclear rim distribution observed in mock-infected cells and cells during the early stage of infection, a portion of the GFP-lamin B showed altered localization and seemed diffuse at the nuclear rim at 18 h p.i., and distributed in the ring zone within the nucleus beginning at 24 h p.i. in virus-infected cells (Fig. 3A). Thus, the nuclear lamina appears to be partially disrupted during baculovirus infection. This virus-induced altered localization of GFP-lamin B indicated the involvement of GFP-lamin B in the virus-host interaction. The distribution of GFP-lamin B within the nucleus was further confirmed by IEM (Fig. 4). Based on the association between GFP-lamin B and nucleocapsids and the edges of the electron-dense stromal mattes of the VS, a certain portion of GFP-lamin B is released from the nuclear lamina and binds to the non-membranous structure. These results further confirm the disruption of the nuclear lamina, which appears to be a dynamic event because the diffuse region quickly recovered, as observed by live cell confocal imaging (Fig. 4C). Moreover, the diffuse distribution of GFP-lamin B was observed only in a restricted area of the nuclear rim during infection, and large-scale dismantling, which occurs during mitosis^41^, was not observed. This observation suggests a sophisticated manipulation of nuclear lamina dynamics by baculoviruses to promote the nuclear egress of nucleocapsids while maintaining the majority of the lamina structure to ensure nuclear integrity, as ODVs develop within the nucleus during the late phase of infection^15^.

During the preparation of this manuscript, Wei *et al*.^32^ cloned a *lamin* gene in the Sf9 cell line. We compared the amino acid sequence of Sf9 lamin with *Drosophila* lamin B and found that all the experimentally confirmed phosphorylation sites for *Drosophila* lamin B are fully conserved between these two lamins (data not shown). Wei *et al*.^32^ also described alterations to the Sf9 nuclear lamina during AcMNPV infection based on immunofluorescence analyses. However, they did not find remarkable structure alternations. First of all, diffuse localization of nuclear rim lamins was not detected. This discrepancy may be attributable to the different time points selected. According to our results, diffuse localization of lamins at the nuclear rim was easily observed at 18 h p.i., at which time approximately 67.5% of cells exhibited this phenomenon. This percentage decreased as infection progressed, to 33.3% at 24 h p.i. and 13.2% at 36 h p.i. Wei *et al*.^32^ detected lamin localization beginning at 48 h p.i., and therefore, diffuse localization of lamins may not have been observed. In addition, the authors did not observe lamin redistribution within the nuclei of infected cells, unlike the results obtained in our study. This inconsistency is potentially due to the different methods used for fluorescence detection in the two studies: lamin B localization was observed by detecting immunofluorescence associated with the ADL67 antibody, which binds lamin B, in the report by Wei *et al*.^32^, but localization was monitored based on GFP autofluorescence in the present analysis. GFP autofluorescence intensity may be stronger than that of the immunofluorescent signal associated with the ADL67 antibody. As the GFP autofluorescence intensity within the nucleus is quite weak compared with Ac76 (Fig. 3A), it may not be detectable using the ADL67 antibody in immunofluorescence analyses.

IEM revealed the localization of GFP-lamin B to virus-induced intranuclear microvesicles, ODV envelopes and nucleocapsids (Fig. 4), consistent with the confocal microscopy results which showed the distribution of GFP-lamin B at the ring zone (Fig. 3A). The majority of B-type lamins remain associated with the INM during mitosis due to isoprenylation modifications or specific interactions with the lamin B receptor^42^. The formation of intranuclear microvesicles, which serve as precursors of the ODV envelope, is thought to be the result of budding of discrete regions of the INM into the nucleoplasm^15^. Thus, we speculate that a fraction of lamin B is released from the lamina while the rest remains associated with the INM, localizes to intranuclear microvesicles via budding of the INM into the nucleoplasm and is subsequently associated with the ODV envelopes when the intranuclear microvesicles assemble as ODV envelopes during baculovirus infection.

In addition to the nuclear membrane, lamins bind directly to nucleic acids and histones and thus bind chromatin^43–45^. Lamin-chromatin interactions have been conserved throughout evolution, as *C. elegans*, *Drosophila*, *Xenopus* and mammalian lamins interact with decondensed sperm chromatin, assembled chromatin or isolated mitotic chromosomes from different species *in vitro* ^44^. Protamines, which may have evolved from H1-like histones, replace histones and form stable complexes with DNA to condense the spermatid genome into a genetically inactive state during spermatogenesis^46^. P6.9, a protamine-like protein encoded by baculoviruses, is thought to bind to and condense baculoviral DNA for packaging into capsids, during which P6.9 is dephosphorylated^47^. C-terminal unphosphorylated forms of P6.9 primarily localize to the edges of the electron-dense stromal mattes of the VS as well as nucleocapsids^48^. Notably, GFP-lamin B was observed associated with the edges of the electron-dense stromal mattes of the VS and the nucleocapsids at the periphery of the stromal mattes, and this distribution pattern is quite similar to that of C-terminal unphosphorylated P6.9. GFP-lamin B may bind P6.9 or viral DNA and thus associate with nucleocapsids and the edges of the electron-dense stromal mattes of the VS. Further studies are required to determine whether lamin B associates with P6.9 and plays a role in nucleocapsid assembly via this association.

Lamina disassembly is associated with lamin phosphorylation by CDK1 during mitosis^8^ and by PKCδ during apoptosis^7^. During herpesvirus infection, the nuclear lamina is disrupted in a nuclear-envelopment-complex (NEC)-dependent manner through multiple mechanisms, and both viral and cellular kinases participate in lamin phosphorylation^49^. Thus, we infer that the lamina disruption during baculovirus infection may also correlate with lamin phosphorylation. GFP-lamin B phosphorylation was detected by performing Phos-tag SDS-PAGE, and phosphorylated lamin B was detected beginning at 12 h p.i. and maintained through 48 h p.i. Lamin phosphorylation is required to drive the disassembly of the nuclear lamina or to regulate lamin import into the nucleus^2^. The majority of host protein synthesis is halted during baculovirus infection^50–52^. According to a recent study, the total amount of lamin B does not increase during baculovirus infection^32^. Thus, the lamin B phosphorylation detected in the present study most likely drives nuclear lamina disassembly rather than regulates lamin import into the nucleus. Moreover, consistent with the IEM data in which GFP-lamin B was distributed within the nuclei of infected cells at 12 h p.i. (Fig. 4), a virally induced increase in GFP-lamin B phosphorylation was detected as early as 12 h p.i., further suggesting that lamina disruption may be regulated by lamin phosphorylation during baculovirus infection. These data also implied that lamina disruption occurs before the onset of BV production because the onset of detectable BV production occurs between 14 and 15 h p.i. in AcMNPV-infected cells^53^. This result is consistent with our hypothesis that disruption of the nuclear lamina is required for the nucleocapsid to gain access to the INM for nuclear egress and subsequent budding at the cytoplasmic membrane^54^. Given that the intranuclear microvesicles are thought to be derived from the INM and the formation of the intranuclear microvesicles requires a more fluid nuclear membrane^15^, it cannot be excluded that the lamina disruption may also be required for intranuclear microvesicle formation. Several baculovirus-encoded genes are involved in the nuclear egress of nucleocapsids, including *gp41* ^55^, *exon*0^56^, *ac66* ^57^, *p48* ^58^, and *ac93* ^54^. Notably, *ac93* is required for both the nuclear egress of nucleocapsids and intranuclear microvesicle formation^54^, indicating these two processes may share some common steps. It is necessary to determine whether these genes participate in the disruption of the nuclear lamina. The mechanisms underlying baculovirus-induced lamina disruption are the subject of ongoing investigations.

## Methods

### Cells and viruses

Sf9 cells were maintained at 27 °C in Grace’s insect medium (Invitrogen Life Technologies) supplemented with 10% fetal bovine serum (Invitrogen Life Technologies), 100 μg/ml penicillin, and 30 μg/ml streptomycin. The recombinant wild type virus with a *gfp* reporter gene, vAcWT, was constructed by inserting the *polyhedrin* and *gfp* genes into the *polyhedrin* locus of the bacmid bMON14272^59^. BV titers were determined by performing a 50% TCID_50_ endpoint dilution assay in Sf9 cells or Sf9-L cells.

### Construction of viruses

To construct the FLAG-tagged *ac76* repair bacmid, a fragment containing the *ac76* promoter sequence and ORF tagged with the FLAG coding sequence at the 3′ end was PCR-amplified from the recombinant plasmid pIB-Ac76:FLAG^29^ with the primers ac76-F (5′-GGGGTACCATGAATTTATATTTGTTGTTGGGCG-3′ [the EcoRI site is underlined]) and ac76:FLAG-R (5′-AACTGCAGTCACTTATCGTCGTCATCCTTGTAAT-3′ [the PstI site is underlined]). The linear fragment was cloned into the pFB1-ph-ac76-gfp vector^31^ to create the donor plasmid pFB1-Ac76:FLAG-PH. Both the FLAG-tagged *ac76* and *polyhedrin* genes were finally transposed into the *polyhedrin* locus of the *ac76* knockout bacmid bAc76KO^31^ to generate the recombinant bacmid vAc76:FLAG via site-specific transposition as previously described^31^.

To compare viral replication between Sf9-L and Sf9 cells, vAcWT-mCh was constructed by inserting the *polyhedrin* and *mCherry* genes into the *polyhedrin* locus of the bacmid bMON14272. A donor plasmid was constructed using a simple cloning method as previously described with minor modifications^60, 61^. Briefly, a linear fragment containing the *mCherry* ORF and the sequences flanking the *gfp* gene in the vector backbone pFB1-PH-GFP^30^ at each end was amplified from the pCMV-mCherry plasmid (Clontech) with the forward primer US-mCherry-F (5′-GTTGACACTGGCGGCGACAAGATCGTGAACAACCAAGTGACTATGGTGAGCAAGGGCGAGG-3′ [the sequence upstream of *gfp* is underlined]) and the reverse primer DS-mCherry-R (5′-CTTCTCGACAAGCTTGGTACCTTATTTGTATAGTTCATCCATGCCATGTTACTTGTACAGCTCGTCCAT-3′ [the sequence downstream of *gfp* is underlined]). Prolonged overlap extension PCR (POE-PCR) was performed using the amplified linear fragments as mega primers and pFB1-PH-GFP as the template under the following conditions: 98 °C for 5 min, followed by 35 cycles of 98 °C for 10 s, 60 °C for 10 s, and 72 °C at a rate of 2 kb/min, with a final step at 72 °C for 10 min. Next, 20 U of DpnI restriction enzyme (New England Biolabs) was added to the PCR product and incubated for 3 h to digest the parental plasmids. The resulting mixture was transformed into chemically competent DH5α cells to generate the plasmid pFB1-PH-mCherry. Correctly sequenced pFB1-PH-mCherry was transformed into electrocompetent DH10B cells harboring the pMON7124 helper plasmid and bMON14272 to generate the bacmid vAcWT-mCh via site-specific transposition.

The above recombinants were verified by PCR analysis. Bacmid DNAs were isolated with a Qiagen Large-Construct Kit (Qiagen), and the concentrations were quantified by determining the optical density.

### Generation of a clonal cell line stably expressing GFP-tagged lamin B

To monitor the localization of lamin B in Sf9 cells, we generated a stable clonal Sf9 cell line constitutively expressing GFP-tagged lamin B of *Drosophila melanogaster* ^27, 62^. The *gfp* ORF lacking a termination codon was PCR-amplified from the vAcWT viral sequence with the primers GFP-F (5′-AAGCTTCGCCACCATGGTGAGCAAG-3′ [the HindIII site is underlined]; CGCCACC was added as a Kozak sequence) and GFP-R (5′-GGTACCCTTGTACAGCTCGTCCATG-3′ [the KpnI site is underlined]). The *lamin B* ORF was PCR-amplified from the plasmid VB465 (donated by Dr. Nico Stuurman, University of California, San Francisco, California, USA) with the primers LmnB-F (5′-GGTACCATGTCGAGCAAATCCCGACG-3′ [the KpnI site is underlined]) and LmnB-R (5′-GAATTCTTACATAATGGCGCACTTCTCGT-3′ [the EcoRI site is underlined]). The resulting fragments were cloned into the expression vector pIB/V5-His (Invitrogen Life Technologies), which contains the *Orgyia pseudotsugata multiple nucleopolyhedrovirus* (OpMNPV) *ie2* immediate-early promoter^63–65^ and the blasticidin-resistant gene, to generate the pIB-GFP:LmnB expression plasmid. The construct was verified by PCR analysis and DNA sequencing.

To generate the GFP-lamin B cell line, adherent Sf9 cell monolayers (5 × 10^5^ cells/35-mm-diameter dish) were transfected with 2 μg of pIB-GFP:LmnB using Cellfectin II reagent (Invitrogen Life Technologies). After culturing for 36 h, the complete medium was replaced with selective medium containing 60 μg/ml blasticidin (Invitrogen Life Technologies), a concentration previously identified from a kill curve according to the manufacturer’s instructions. The selective medium was replaced every 3 days until GFP-positive cellular colonies formed.

Clonal cell lines were isolated using Millicell hanging cell culture inserts (0.33 cm^2^, 1-μm porosity; Millipore) according to the manufacturer’s instructions. Millicell hanging cell culture inserts were loaded in 24-well culture plates seeded with GFP-positive colonies (2 × 10^5^/well) in selective medium containing 30 μg/ml blasticidin, followed by the addition of 100 μl of complete medium to each insert. After culturing at 27 °C for 24 h, the medium in the insert was discarded, and each insert was seeded with one GFP-lamin B-positive cell and cultured in complete medium for 24 h, followed by culturing in selective medium. The selective medium was replaced every 4 days until a monolayer formed. Simultaneously, cells in 24-well plates were passaged and incubated in selective medium. Confluent monolayers on inserts were subcultured in larger plates and maintained with 30 μg/ml blasticidin. One of the clonal cell lines was selected for further analysis and designated Sf9-L.

### Time course analysis of total GFP-lamin B

Sf9-L cells (1 × 10^6^ cells/35-mm-diameter dish) were infected with vAcWT at a multiplicity of infection (MOI) of 10. At the indicated time points p.i., the cells were washed twice with phosphate-buffered saline (PBS) and collected via centrifugation at 1,000 × *g* for 10 min. The pelleted cells were lysed on ice for 15 min in radio-immunoprecipitation assay (RIPA) buffer (25 mM Tris-HCl [pH 7.6], 150 mM NaCl, 1% NP-40, 1% sodium deoxycholate, 0.1% sodium dodecyl sulfate [SDS]; Thermo Scientific) supplemented with protease inhibitor cocktail tablets (one tablet/25 ml; Roche) and then mixed with an equal volume of 2 × protein sample buffer. Equal volumes of each sample were resolved by SDS-10% PAGE, electrophoretically transferred to polyvinylidene difluoride membranes (Millipore), and probed with mouse monoclonal anti-GFP (1:2,000; Abmart) or anti-actin (1:2,000; Abmart) antibodies according to the manufacturer’s instructions. Specific proteins were detected with a goat anti-mouse horseradish peroxidase (HRP)-conjugated antibody (1:5,000; Pierce) and enhanced chemiluminescence reagents (ECL; Amersham Biosciences) on standard X-ray film (Kodak).

### Detection of GFP-lamin B phosphorylation

Sf9-L cells (1.5 × 10^6^) were infected with vAcWT at an MOI of 10. At the indicated time points p.i., the cells were washed twice with PBS and collected via centrifugation at 1,000 × *g* for 10 min. Equal numbers of pelleted cells were lysed on ice for 30 min in immunoprecipitation (IP) buffer (20 mM Tris [PH 7.5], 150 mM NaCl, 1% Triton X-100; Beyotime) supplemented with 0.15% SDS, protease inhibitor cocktail tablets (one tablet/25 ml; Roche), 1 mM PMSF (Sigma), 20 mM NaF (Sigma), Phosphatase Inhibitor Cocktail Set IV (1:50; Merck) and 1 mM MnCl_2_. Cell lysates were mixed with 3 × protein sample loading buffer (3.9 ml of 0.5 M Tris-HCl [pH 6.8], 0.6 g of SDS, 3.0 ml of 100% glycerol, 1.5 ml of 2-mercaptoethanol [2-ME], 1.5 mg of 0.02% bromophenol blue) according to the manufacturer’s instructions (Wako) and boiled at 100 °C for 10 min. The samples were resolved by Mn^2+^-Phos-tag SDS-8% PAGE containing 25 μM Phos-tag and 0.1 mM MnCl_2_ as previously described^66^ at a constant current of 25 mA at 10 °C for 7 h. After electrophoresis, the polyacrylamide gel was washed three times (40 min each) with transfer buffer containing 35 mM ethylene diamine tetraacetic acid (EDTA), followed by two washes (30 min each) with transfer buffer without EDTA according to the manufacturer’s instructions (Wako) with some modifications. Subsequently, the proteins were electroblotted onto polyvinylidene difluoride membranes (Millipore) at a constant current (270 mA) for 4 h. Immunoblotting was performed with a mouse monoclonal anti-GFP antibody (1:2,000; Abmart) as described above.

### Immunofluorescence and live confocal microscopy

Sf9-L cells (5 × 10^5^) were seeded into 35-mm glass-bottom culture dishes (MatTek) and mock-infected or infected with vAc76:FLAG at an MOI of 10. At the indicated time points p.i., the cells were processed for immunofluorescence microscopy as previously described^29^. For immunolabeling, mouse monoclonal anti-FLAG (1:200; Abmart) or ADL67 to detect lamin B (1:50)^62^ was used as the primary antibody, and donkey anti-mouse IgG conjugated to Alexa Fluor 555 (1:400; Invitrogen/Molecular Probes) was used as the secondary antibody. Prior to analysis, the labeled cells were stained with Hoechst 33342 (Invitrogen/Molecular Probes), followed by three washes with PBS. Images were collected with a Zeiss LSM 780 confocal laser scanning microscope.

For live fluorescence imaging, Sf9-L cells (5 × 10^5^) were infected with vAc76:FLAG and viewed with a confocal microscope as described above.

### IEM

Sf9-L cells (1.5 × 10^6^) were infected with vAc76:FLAG at an MOI of 10. At the indicated time points p.i., cells were pelleted at 800 × *g* for 10 min and prepared for IEM with LR White resin (Ted Pella, Inc.) as previously described^29^. Immunolabeling was performed with a mouse monoclonal anti-GFP antibody (1:200; Abmart), followed by incubation with goat anti-mouse IgG conjugated to 10-nm gold particles as the secondary antibody (1:50; Sigma). The samples were visualized with a JEOL JEM-1400 TEM at an accelerating voltage of 120 kV.

## References

[1] Burke B, Stewart CL (2013). The nuclear lamins: flexibility in function. Nat. Rev. Mol. Cell Biol..

[2] Dechat T, Adam SA, Taimen P, Shimi T, Goldman RD (2010). Nuclear lamins. Cold Spring Harb. Perspect. Biol..

[3] Gruenbaum Y, Margalit A, Goldman RD, Shumaker DK, Wilson KL (2005). The nuclear lamina comes of age. Nat. Rev. Mol. Cell Biol..

[4] Lee CP, Chen MR (2010). Escape of herpesviruses from the nucleus. Rev. Med. Virol..

[5] Broers JL, Ramaekers FC, Bonne G, Yaou RB, Hutchison CJ (2006). Nuclear lamins: laminopathies and their role in premature ageing. Physiol. Rev..

[6] Goldberg MW, Fiserova J, Huttenlauch I, Stick R (2008). A new model for nuclear lamina organization. Biochem. Soc. Trans..

[7] Cross T (2000). PKC-delta is an apoptotic lamin kinase. Oncogene.

[8] Nigg EA (1992). Assembly-disassembly of the nuclear lamina. Curr. Opin. Cell Biol..

[9] Johnson DC, Baines JD (2011). Herpesviruses remodel host membranes for virus egress. Nat. Rev. Microbiol..

[10] Mettenleiter TC, Muller F, Granzow H, Klupp BG (2013). The way out: what we know and do not know about herpesvirus nuclear egress. Cell Microbiol..

[11] Mettenleiter TC (2002). Herpesvirus assembly and egress. J. Virol..

[12] Klupp BG (2007). Vesicle formation from the nuclear membrane is induced by coexpression of two conserved herpesvirus proteins. Proc. Natl. Acad. Sci. USA.

[13] Herniou EA, Olszewski JA, Cory JS, O’Reilly DR (2003). The genome sequence and evolution of baculoviruses. Annu. Rev. Entomol..

[14] Rohrmann, G. F. *Baculovirus molecular biology* (ed. George F. Rohrmann) 3rd edn (US National Center for Biotechnology Information, 2013). 24479205

[15] Braunagel SC, Summers MD (2007). Molecular biology of the baculovirus occlusion-derived virus envelope. Curr. Drug Targets.

[16] Jehle JA (2006). On the classification and nomenclature of baculoviruses: a proposal for revision. Arch. Virol..

[17] Braunagel SC, Russell WK, Rosas-Acosta G, Russell DH, Summers MD (2003). Determination of the protein composition of the occlusion-derived virus of *Autographa californica* nucleopolyhedrovirus. Proc. Natl. Acad. Sci. USA.

[18] Deng F (2007). Proteomics analysis of *Helicoverpa armigera* single nucleocapsid nucleopolyhedrovirus identified two new occlusion-derived virus-associated proteins, HA44 and HA100. J. Virol..

[19] Hou D (2013). Comparative proteomics reveal fundamental structural and functional differences between the two progeny phenotypes of a baculovirus. J. Virol..

[20] Wang R (2010). Proteomics of the *Autographa californica* nucleopolyhedrovirus budded virions. J. Virol..

[21] Li Z, Blissard GW (2010). Baculovirus GP64 disulfide bonds: the intermolecular disulfide bond of *Autographa californica* multicapsid nucleopolyhedrovirus GP64 is not essential for membrane fusion and virion budding. J. Virol..

[22] van Oers MM, Vlak JM (2007). Baculovirus genomics. Curr. Drug Targets.

[23] Slack J, Arif BM (2007). The baculoviruses occlusion-derived virus: virion structure and function. Adv. Virus Res..

[24] Williams, G. & Faulkner, P. Cytological changes and viral morphogenesis during baculovirus infection in *The baculoviruses* (ed. Lois K. Miller) pp 61–107 (Springer US, 1997).

[25] Knudson DL, Harrap KA (1975). Replication of nuclear polyhedrosis virus in a continuous cell culture of *Spodoptera frugiperda*: microscopy study of the sequence of events of the virus infection. J. Virol..

[26] Ge JQ (2008). Characterization of a nucleopolyhedrovirus with a deletion of the baculovirus core gene *Bm67*. J. Gen. Virol..

[27] Klapper M (1997). Assembly of A-and B-type lamins studied *in vivo* with the baculovirus system. J. Cell. Sci..

[28] Braunagel SC (2004). Trafficking of ODV-E66 is mediated via a sorting motif and other viral proteins: facilitated trafficking to the inner nuclear membrane. Proc. Natl. Acad. Sci. USA.

[29] Wei D (2014). *Autographa californica* Nucleopolyhedrovirus Ac76: a dimeric type II integral membrane protein that contains an inner nuclear membrane-sorting motif. J. Virol..

[30] Wu W (2006). *Autographa californica* multiple nucleopolyhedrovirus nucleocapsid assembly is interrupted upon deletion of the *38K* gene. J. Virol..

[31] Hu Z (2010). *Autographa californica* multiple nucleopolyhedrovirus *ac76* is involved in intranuclear microvesicle formation. J. Virol..

[32] Wei W (2016). Cloning and characterization of Sf9 cell lamin and the lamin conformational changes during *Autographa californica* multiple nucleopolyhedrovirus infection. Viruses.

[33] Gerace L, Blobel G (1980). The nuclear envelope lamina is reversibly depolymerized during mitosis. Cell.

[34] Guttinger S, Laurell E, Kutay U (2009). Orchestrating nuclear envelope disassembly and reassembly during mitosis. Nat. Rev. Mol. Cell Biol..

[35] Kinoshita E, Kinoshita-Kikuta E, Takiyama K, Koike T (2006). Phosphate-binding tag, a new tool to visualize phosphorylated proteins. Mol. Cell Proteomics.

[36] Vanarsdall AL, Mikhailov VS, Rohrmann GF (2007). Characterization of a baculovirus lacking the DBP (DNA-binding protein) gene. Virology.

[37] Machowska, M., Piekarowicz, K. & Rzepecki, R. Regulation of lamin properties and functions: does phosphorylation do it all? *Open Biol*. **5**, doi:10.1098/rsob.150094 (2015). 10.1098/rsob.150094 PMC4680568 26581574

[38] Mou F, Forest T, Baines JD (2007). US3 of herpes simplex virus type 1 encodes a promiscuous protein kinase that phosphorylates and alters localization of lamin A/C in infected cells. J. Virol..

[39] Broers JL (1999). Dynamics of the nuclear lamina as monitored by GFP-tagged A-type lamins. J. Cell. Sci..

[40] Scott ES, O’Hare P (2001). Fate of the inner nuclear membrane protein lamin B receptor and nuclear lamins in herpes simplex virus type 1 infection. J. Virol..

[41] Margalit A, Vlcek S, Gruenbaum Y, Foisner R (2005). Breaking and making of the nuclear envelope. J. Cell. Biochem..

[42] Meier J, Georgatos SD (1994). Type B lamins remain associated with the integral nuclear envelope protein p58 during mitosis: implications for nuclear reassembly. EMBO J..

[43] Goldberg M (1999). The tail domain of lamin Dm_0_ binds histones H2A and H2B. Proc. Natl. Acad. Sci. USA.

[44] Prokocimer M (2009). Nuclear lamins: key regulators of nuclear structure and activities. J. Cell. Mol. Med..

[45] Taniura H, Glass C, Gerace L (1995). A chromatin binding site in the tail domain of nuclear lamins that interacts with core histones. J. Cell Biol..

[46] Balhorn R (2007). The protamine family of sperm nuclear proteins. Genome Biol..

[47] Tweeten KA, Bulla LA, Consigli RA (1980). Characterization of an extremely basic protein derived from granulosis virus nucleocapsids. J. Virol..

[48] Liu X (2012). Distribution and phosphorylation of the basic protein P6.9 of *Autographa californica* nucleopolyhedrovirus. J. Virol..

[49] Park R, Baines JD (2006). Herpes simplex virus type 1 infection induces activation and recruitment of protein kinase C to the nuclear membrane and increased phosphorylation of lamin B. J. Virol..

[50] Chen YR (2013). The transcriptome of the baculovirus *Autographa californica* multiple nucleopolyhedrovirus in *Trichoplusia ni* cells. J. Virol..

[51] Du X, Thiem SM (1997). Responses of insect cells to baculovirus infection: protein synthesis shutdown and apoptosis. J. Virol..

[52] Schultz KL, Friesen PD (2009). Baculovirus DNA replication-specific expression factors trigger apoptosis and shutoff of host protein synthesis during infection. J. Virol..

[53] Milks ML, Washburn JO, Willis LG, Volkman LE, Theilmann DA (2003). Deletion of *pe38* attenuates AcMNPV genome replication, budded virus production, and virulence in *heliothis virescens*. Virology.

[54] Yuan M (2011). Identification of *Autographa californica* nucleopolyhedrovirus *ac93* as a core gene and its requirement for intranuclear microvesicle formation and nuclear egress of nucleocapsids. J. Virol..

[55] Olszewski J, Miller LK (1997). A role for baculovirus GP41 in budded virus production. Virology.

[56] Fang M, Dai X, Theilmann DA (2007). *Autographa californica* multiple nucleopolyhedrovirus EXON0 (ORF141) is required for efficient egress of nucleocapsids from the nucleus. J. Virol..

[57] Ke J, Wang J, Deng R, Wang X (2008). *Autographa californica* multiple nucleopolyhedrovirus *ac66* is required for the efficient egress of nucleocapsids from the nucleus, general synthesis of preoccluded virions and occlusion body formation. Virology.

[58] Yuan M (2008). A highly conserved baculovirus gene *p48* (*ac103*) is essential for BV production and ODV envelopment. Virology.

[59] Cai Y (2012). An *ac34* deletion mutant of *Autographa californica* nucleopolyhedrovirus exhibits delayed late gene expression and a lack of virulence *in vivo*. J. Virol..

[60] Huang Z (2015). Introduction of temperature-sensitive helper and donor plasmids into Bac-to-Bac baculovirus expression systems. Virol. Sin..

[61] You C, Zhang XZ, Zhang YH (2012). Simple cloning via direct transformation of PCR product (DNA Multimer) to *Escherichia coli* and *Bacillus subtilis*. Appl. Environ. Microbiol..

[62] Stuurman N, Maus N, Fisher PA (1995). Interphase phosphorylation of the *Drosophila* nuclear lamin site-mapping using a monoclonal antibody. J. Cell Sci..

[63] Hegedus DD, Pfeifer TA, Hendry J, Theilmann DA, Grigliatti TA (1998). A series of broad host range shuttle vectors for constitutive and inducible expression of heterologous proteins in insect cell lines. Gene.

[64] Theilmann DA, Stewart S (1992). Tandemly repeated sequence at the 3′ end of the *IE-2* gene of the baculovirus *Orgyia pseudotsugata* multicapsid nuclear polyhedrosis virus is an enhancer element. Virology.

[65] Theilmann DA, Stewart S (1992). Molecular analysis of the trans-activating *IE-2* gene of *Orgyia pseudotsugata* multicapsid nuclear polyhedrosis virus. Virology.

[66] Kinoshita-Kikuta E, Kinoshita E, Matsuda A, Koike T (2014). Tips on improving the efficiency of electrotransfer of target proteins from Phos-tag SDS-PAGE gel. Proteomics.

